# Comparing embedded virtual reality combat to traditional warm-ups on fencing performance and fatigue: Protocol for a randomized crossover trial

**DOI:** 10.1016/j.jsampl.2026.100144

**Published:** 2026-06-13

**Authors:** Chao Bian, Suzanna Russell, Kevin De Pauw, Rafael Kons, Sidney Grosprêtre, Špela Bogataj, Bart Roelands

**Affiliations:** a Human Physiology and Sports Physiotherapy Research Group (MFYS), Vrije Universiteit Brussel, Brussels, Belgium; b BruBotics, Vrije Universiteit Brussel, Brussels, Belgium; c Department of Physical Education, Federal University of Bahia, Salvador, Brazil; d Laboratory C3S, Culture Sport Health and Society, Sport and Performance Department, University of Franche-Comté, Besançon, France; e Institut Universitaire de France (IUF), Paris, France; f Department of Nephrology, University Medical Center Ljubljana, Ljubljana, Slovenia; g Faculty of Sport, University of Ljubljana, Ljubljana, Slovenia; h Laboratory of Sports and Nutrition Research, Riga Stradins University, Riga, Latvia

**Keywords:** Mental fatigue, Cognitive priming, Psychomotor performance, Combat sports, Ecological validity, Psychophysiological response

## Abstract

**Background:**

Mental fatigue has been observed to increase early and accumulate during fencing competition days. Traditional warm-up structures in fencing, including coach-led one-on-one combat (TC), always require a physical sparring partner. Virtual reality combat (VC) can engage athletes in an immersive, entertaining sports environment with specific motor-cognitive stimuli to enhance training and mental preparation, which may facilitate a quality warm-up.

**Aims:**

This study will compare the effects of VC with those of TC in the specialized warm-up section, on fencing-specific performance and fencers' fatigue levels.

**Methods:**

This study will be a within-subjects, randomized crossover design. It has been ethically approved and registered. Informed consent will be obtained from all participants. Twenty Epee fencers will be recruited and matched for gender and fencing level. The fencers' pair will be randomly assigned in a counterbalanced order to commence the experimental condition with VC or the control condition with TC first. Standardized measures of mental and physical fatigue, and motor-cognitive performance will be conducted pre- and post-warm-ups.

**Analysis:**

Linear mixed-effects models will be used for time, condition, and their interaction effects. Other subjective perceptions after VC/TC and heart rate responses to VC/TC will be collected and compared between conditions via paired samples t-tests.

**Discussion:**

This is the first time the virtual reality technology will be integrated into a traditional fencing warm-up to explore the feasibility of virtual combat as an on-field alternative. The practice-oriented study seeks to inform pre-competition warm-up structures, management of mental fatigue, and subsequent performance enhancement in real-world combat sports.

**KEY POINTS:**

**What the study is likely to add/Possible implications:**

• By assessing subjective and objective indicators of mental and physical fatigue, and an ecologically valid fencing-specific test, this study will scientifically verify the effects of a traditional warm-up structure on subsequent performance and fencers' fatigue level.
• For the first time, the study will explore a fencing-themed virtual combat being integrated into a traditional fencing warm-up procedure as a feasible, on-field alternative.
• Applying a brief virtual reality exergame before competition can engage athletes in an entertaining scenario that provides specific motor-cognitive stimuli, which may buffer fatigue perception and facilitate mental preparation.
• The practice-oriented study may inform redesign of warm-up structures, management of mental fatigue, and subsequent performance enhancement in real-world combat sports.

## 1 Introduction

Fencing is an open-skilled combat sport characterized by high intermittency [1], with short bursts of sword fighting and frequent rest intervals [2]. This leads to non-maximal physiological demands and relatively minor physical fatigue [3]. Fencing, however, requires the allocation of mental resources and perceptual-cognitive skills (e.g., sustained attention, information processing, response inhibition) for performance maintenance and success [[4], [5], [6]]. The prolonged interaction between motor and cognitive demands in the congested competition format (i.e., featuring consecutive preliminary and elimination bouts on the same day) can cause the onset of mental fatigue in fencers [7,8]. Longitudinal tracking during a national championship revealed elevated perceived mental fatigue and slower reaction time (RT) after competitions, suggesting a detrimental effect of mental fatigue on fencing performance [8]. During the period, the daily baseline mental fatigue fluctuated and peaked on competition days [8]. Furthermore, in the early stage of the consecutive bout progression, mental fatigue tended to accumulate faster [8]. This highlights the importance of mental fatigue management since the competition mornings [9]. The pre-competition preparation period, which may first determine the initial state of mental fatigue and subsequent competitive performance, emerges as a valuable time window worthy of attention.

Pre-competition warm-up is vital to attain optimal performance by evoking temperature, metabolic, neural, and psychological-related effects [10]. Meanwhile, warm-up strategies across sports are developed largely through a trial-and-error basis and the coach's experience [10]. Typically, a pre-competition warm-up protocol in fencing includes a generalized physical section, and the coach or sparring partner-led, technical/tactical drills and combat in a specialized section that requires a 1:1 personnel configuration to meet its high-level, competition-like demands. Yet, scientific evidence is lacking regarding the effect of such a warm-up on subsequent fencing performance. Moreover, mental fatigue could arguably build up due to the specific motor-cognitive demands from the warm-up combat, exacerbating its accumulation throughout the forthcoming competition day [9]. Further optimization of the warm-up could potentially enhance the performance [11,12] but lower the risk of prematurely elevating mental fatigue in fencers.

From this perspective, Virtual Reality (VR) technology holds potential as a controlled and adaptable warm-up tool, enabling the simulation of sport-specific perceptual and decision-making demands while limiting unnecessary physical and cognitive load. Indeed, VR is already recognized as a valuable tool in sports performance contexts, offering applications for training, skill learning, and mental preparation [13]. These applications include targeting sports-specific perceptual-cognitive skills [14]. It immerses users in realistic and interactive scenarios of varying complexity, usually via a head-mounted display and handheld controllers [15]. Recently, exergames have been developed to engage athletes in a virtual sports environment with entertaining tasks mimicking technical drills and even competition [16]. The acute effects of VR exergaming on cerebral activity and cognitive function are documented, for instance, the facilitation of selective attention and inhibitory processes [17]. Accordingly, fencing-themed VR exergames, which are designed for individuals' virtual combat against an avatar, may induce representative motor and cognitive demands concurrently when embedded into warm-ups. Moreover, the brain endurance training (i.e., intermixed physical and cognitive tasks) has been proven to improve performance in fencers [18]. Therefore, a similar benefit may be expected from VR exergaming warm-up, as it employs the same dual-task paradigm.

Importantly, VR is reported to consistently stimulate the autonomic nervous system, as evidenced by increases in heart rate (HR) and skin resistance [19]. It can also lead to reduced vagal activity in the parasympathetic system, as shown by lower HR variability [20], indicating a similar mental fatigue state [21]. Research further suggests that engaging with immersive virtual content increases cognitive demand, potentially causing cognitive decline due to the user's limited attentional resource [22]. Despite being a promising technology that engages the specific motor-cognitive processes that are critical for fencing warm-up and competition-related performance, key concerns remain. It is not yet explored to what extent on-field VR combat can prime the real-world performance, nor what the potential effects are on mental fatigue in fencers. Consequently, this study will compare the effects of virtual combat (using VR) with those of traditional warm-up combat in the specialized warm-up section, on fencing-specific performance and fencers' fatigue levels. This study seeks to inform practitioners to evaluate and optimise warm-up practices. It is particularly relevant when access to an appropriate competitive sparring partner may be limited by availability, or an adapted preparation is required in official competitions, in consideration of the fencer's initial fatigue state.

*Aims*. The primary aim is to compare the acute effects of a 10-min VR combat with a 10-min traditional one-on-one combat during the 20-min specialized warm-up section, on subsequent fencing-specific psychomotor performance and subjective mental and physical fatigue levels in fencers. The secondary aims are: (1) to explore the athlete's psychological experience of the task load, readiness, and enjoyment; (2) to explore the behavioral indicators of mental fatigue and physiological response in the two specialized warm-up conditions.

*Hypotheses*. The hypotheses are: (1) VR combat will benefit the fencing-specific psychomotor performance, while inducing less subjective physical and mental fatigue due to the entertaining game nature. (2) Fencers will perceive less task load in a similar HR response, but more readiness and enjoyment after the embedded VR combat than traditional one-on-one combat. (3) The behavioral indicators of mental fatigue will align with the subjective perceptions of fencers.

## 2 Methods

### 2.1 Study design

**Fig. 1:**
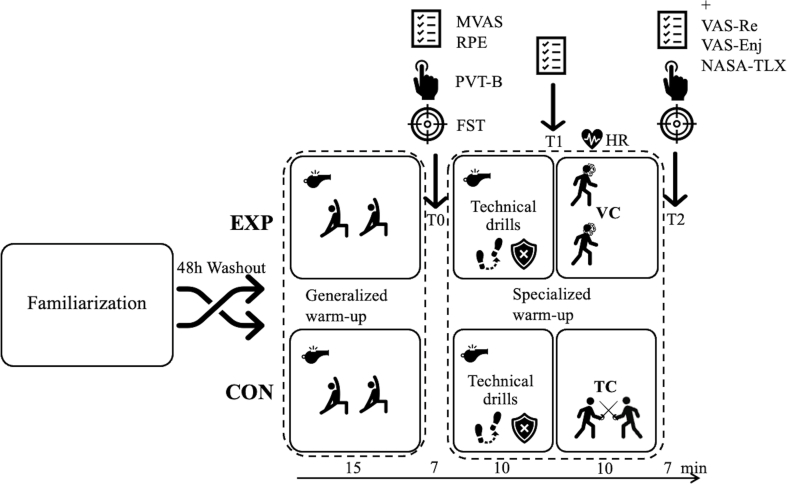
The flow chart and timeline of the study design. EXP, experimental condition; CON, control condition; VC, virtual reality combat; TC, traditional one-on-one combat; MVAS, subjective mental fatigue; RPE, subjective physical fatigue; VAS-Re, readiness to perform; VAS-Enj, enjoyment; PVT-B, brief psychomotor vigilance test; FST, fencing-specific test; NASA-TLX, NASA task load index; HR, heart rate; T, test battery.

The study will employ a within-subjects, randomized crossover design. Participants will be paired (i.e., matched pairs) by the head coach according to registered gender and athletic level. After the familiarization, each matched pair will be randomly assigned in a counterbalanced manner to either commence with the experimental condition (EXP) or the control condition (CON) first. A pre- (T0) and post-test battery (T2) will be conducted for the two conditions (see Fig. 1). Additionally, immediately before VR combat (VC) and traditional one-on-one combat (TC) in the specialized warm-up, the subjective perceptions will be checked again (T1) for standardization.

### 2.2 Ethics and trial status

The study has been approved by the ethics committee of Tsinghua University (THU-01-2026-0021). The study is registered in Open Science Framework (10.17605/OSF.IO/7YCZU) and will be operated following the SPeCIFY guidelines [23].

### 2.3 Participants

To address the primary purpose, the sample size has been calculated via G∗Power 3.1 based on the intended time and condition interaction effect. In a previous study, Harris et al. (2020) used a similar design to compare the training effect in a VR environment with the real-world environment on motor skill performance [24]. They reported a significant, partial eta squared = 0.12 interaction effect. Extracting the effect size value, which corresponds to a Cohen's f = 0.369, with α = 0.05, power (1−β) = 0.90, correlation among repeated measures = 0.5, and non-sphericity correction ε = 1, the minimum required sample size has been determined to be 15. Due to the practical constraints in athletes' recruitment, combined with the possible dropouts (>15%) and the counterbalanced consideration for matched pairs, 20 participants will be recruited to address the first hypothesis.

Active, senior Epee fencers from a university fencing club, featuring a Tier 2–3 (i.e., a trained to well-trained) profile [25], will be considered and screened for eligibility. Eligible participants will receive an email/paper file with an official invitation and informed consent. However, the actual purposes will be masked (i.e., single-blind) by the experience of new VR devices and the internal performance tests in scheduled training sessions. Unblinding procedures will be conducted after completion of data analysis. Following completion of informed consent, online questionnaires will be sent to collect demographic, health, fencing, and lifestyle information. The information will be recorded for further eligibility screening (detailed criteria are seen in Table 1). Participants retain the right to stop participation in the study at any moment.

**Table 1:** The detailed inclusion and exclusion criteria for eligibility.

	Inclusion criteria	Exclusion criteria
1	Aged 18–35 years	Concurrently taking the role of assistant coach/trainer
2	Active Epee fencers from the university club	Training as a Sabre or Foil fencer
3	Being enrolled as a university student	High myopia (no glasses) and red/green color vision defects
4	Understanding concepts both in Chinese and English	Having sleep disorders
5	Being able to provide informed consent individually	Taking any medical treatment during the study
6	Training ∼3 times per week for a competitive purpose	Taking any academic course/examination before tests
7	At least local-level representation in Epee	Having official competitions during the study
8	At least 12 months free from physical injury	Habitual smoker
9	No history of mental illness	Caffeine intake less than 2 h before tests

### 2.4 Procedures

Eligible fencers visit the club pistes 3 times at a fixed slot (e.g., weekly routine training session at 15:00–17:30 to improve adherence), including a familiarization and two conditions (EXP/CON). The washout period will be at least 48h between conditions. The EXP and CON will take approximately 50min. Upon arrival and after preparation, a 15-min standardized general warm-up will be organized by the physical coach before T0. Next, 10min technical drills will be provided by the head coach. In EXP, the two fencers will start VC on their own for 10min. In CON, the paired fencers will conduct TC in the same format for 10min. Immediately before and after the 20-min specialized warm-up (i.e., technical drills and VC/TC), standardized measurements at T0 and T2 will be performed as follows: a 1-min digital questionnaire to collect subjective perceptions, a brief psychomotor vigilance test (PVT-B) to evaluate the reaction performance for 3min, and an Epee fencing-specific test (FST) to assess the reaction speed and lunge attack accuracy of the random targets for another 3min. Between the technical drills and VC/TC (T1), the questionnaire will be prompted to the fencers. Participants will be equipped with HR chest bands (Firstbeat, Garmin, Finland) throughout conditions to check the physiological response to VC/TC.

### 2.5 Field setting

**Fig. 2:**
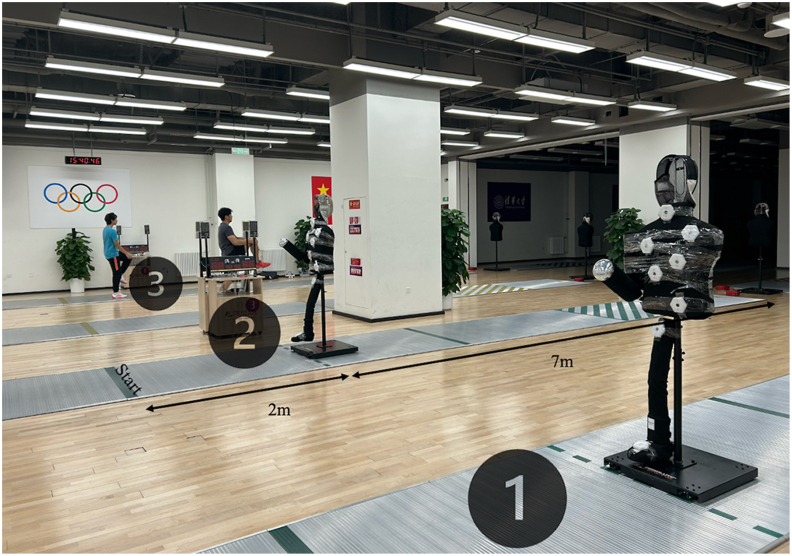
The fencing piste settings and the general fencing club layout.

The study will be performed at Tsinghua University's fencing club (Haidian District, Beijing, China) within a controlled training environment. Three fencing pistes will be prepared as shown in Fig. 2. Two half-pistes (7m) with two fencer-shaped dummies will be set for two FSTs in mirrored layouts. The third piste will be prepared for warm-ups. Particularly, the paired fencers will conduct the 10-min VC separately at each end of the piste (14m).

### 2.6 Familiarization

During the first visit, participants will receive tutorials on wearing VR headsets and HR chest bands properly, as well as manipulating VR controllers to combat. They will be fully familiarized with the related concepts and the entire test battery. The test feedback will be provided for timely guidance and more repeats, if necessary. Particularly, the mental fatigue definition will be given based on Russell et al. (2019) across elite sports contexts [26] and Bian et al. (2025) among Chinese fencers [6], with specific examples of official elimination bouts, examinations, long-distance driving, and prolonged social-media use as the potential inducers.

### 2.7 Generalized warm-up section

The structured 15-min physical warm-up is commonly applied in the club's collective training. This will be executed by the matched-paired fencers together. The light aerobic nature consists of a 2-min jogging, a 3-min shuttle run, a 5-min dynamic stretching, and 5-min physical drills, including stepping, hopping, jumping, side-sliding, agility movements, and throwing the medicine ball against the wall. Immediately after the section, the participants will dress in their fencing clothes.

### 2.8 Specialized warm-up section

#### 2.8.1 Virtual reality combat

**Fig. 3:**
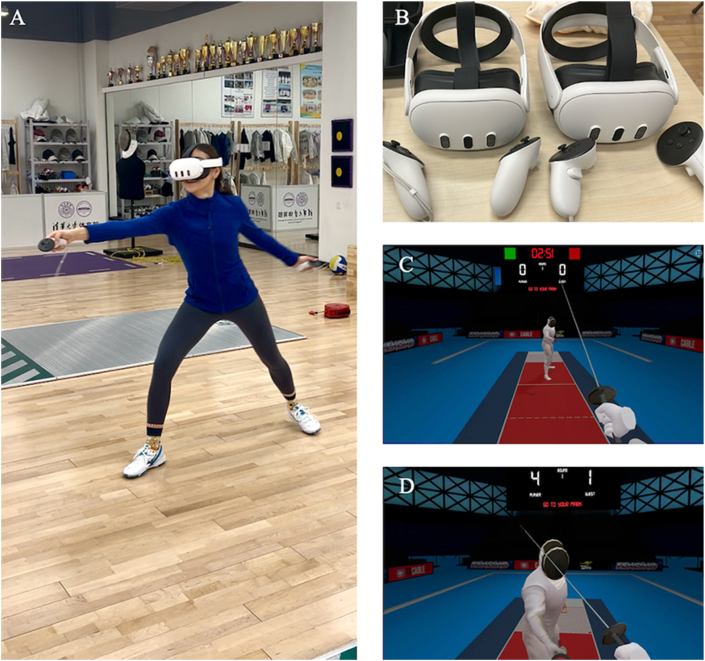
Virtual reality combat. A, participant in the virtual reality combat; B, the virtual reality headsets and controllers; C, the first perspective of the exergame interface; D, the moment the participant engages in combat against the avatar.

Two VR headsets (Quest 3, Meta, US) with the same fencing-themed exergames named "Fencing VR: En Garde Arena" (Abstron Pvt Ltd., Pakistan) will be prepared. One VR headset will be equipped with two controllers (held with both hands) for each of the two fencers (see Fig. 3 A and B). The combat area (i.e., piste) will be framed consistently by the researcher. The Epee competition mode and its virtual environment will be selected for standardization. The VC mirrors the official rules and core cognitive demands of real Epee combat, without any physical impact from the avatar and virtual sword. Participants will compete (against VR as in Fig. 3 C and D) individually for 10min, during which the mean value of the HR response (HRmean) will be calculated.

#### 2.8.2 Traditional one-on-one combat

In official competition, pre-competition preparation after the physical activation typically follows a specialized warm-up section including coach-led technical drills (i.e., footwork, distance, lunge, blade exercises) and a phase of one-on-one combat. This simulation involves high-intensity engagements where fencers execute actual sword fighting. In TC, the paired fencers will be asked to maintain the official attacking tempo to score point by point against each other for 10min, as VC, following the FIE (Fédération Internationale d'Escrime) competition rules. The combat will be controlled under strict timing via an electronic scoring apparatus, which is commonly used in daily training and competitions. The HRmean in TC will also be calculated.

### 2.9 Test battery

#### 2.9.1 Questionnaire

The digital questionnaires will be activated and presented on the tablet at each measurement (i.e., T0, T1, T2). The survey platform (www.wjx.cn) will record the sequence, starting and finishing time automatically. The questions will be answered by reading and sliding the digital scales. The VAS has been validated for fatigue assessments and applied during the real fencing training sessions and competitions for the subjective mental fatigue measures (MVAS) [8]. The participants will slide the marker of the electronic scale to answer "How mentally fatigued do you feel right now?" at T0, T1, and T2. The marker will be initially positioned at "0″ and hidden until participants touch the interface. Two ends of the line will be anchored from "0″ to "100″, indicating "not at all" and "maximum" levels of mental fatigue, respectively. The backend of the online system will record the MVAS by converting it into a 0–100 AU digital form. The digital scale will allow a reset and require confirmation before submission to ensure the value is aligned with the intended response. Meanwhile, after each MVAS measure at T0, T1, and T2, the Rating of Perceived Exertion (RPE) will be measured via the digital CR-10 for subjective physical fatigue [27]. Similarly, at T2, the participants will be further asked to report "readiness to perform" (VAS-Re) and "enjoyment" (VAS-Enj) on VAS following the single-item approach [28], and NASA-TLX (6 subscales on a 21-point Likert scale) based on their experience with the specialized warm-up section. The total estimated completion time for all questionnaires is 1min.

#### 2.9.2 Brief psychomotor vigilance test

The PVT-B has been validated for the reaction performance under a fatigued state and built as a compatible smartphone application [29]. The application (i.e., PVT Research Tool, US) has been utilized for mental fatigue tracking as the behavioral indicators during the real fencing training and competition [8]. Participants will be instructed to gaze at a grey cross on the black screen center and touch the screen as soon as the red, solid circular stimulus appears. They will be required to keep the RT as short as possible, but not to touch the stimulus too soon (i.e., false start or anticipation, display a "Too fast, please wait for the red circle" warning when the RT < 100 ms), or too slow (i.e., timeout, display "time expired" warning when the RT > 10000 ms). The inter-stimulus intervals will vary randomly from 1000 to 4000 ms. After completion, the background system will file each trial's outcomes with the date, starting time, and exact time of every touch. The average RT of the valid touches, the number of valid responses, and false starts will be recorded. Furthermore, the number of lapses (i.e., errors of omission, RT threshold ≥355 ms) defined by Basner et al. [29] will also be applied. For the reaction performance, the accuracy score (RAcc) will be calculated as the ratio of the touches from 100 to 354 ms among all touches. This study will compare the RT and RAcc as behavioral indicators of mental fatigue [8] before and after the specialized warm-up section.

#### 2.9.3 Epee fencing-specific test

**Fig. 4:**
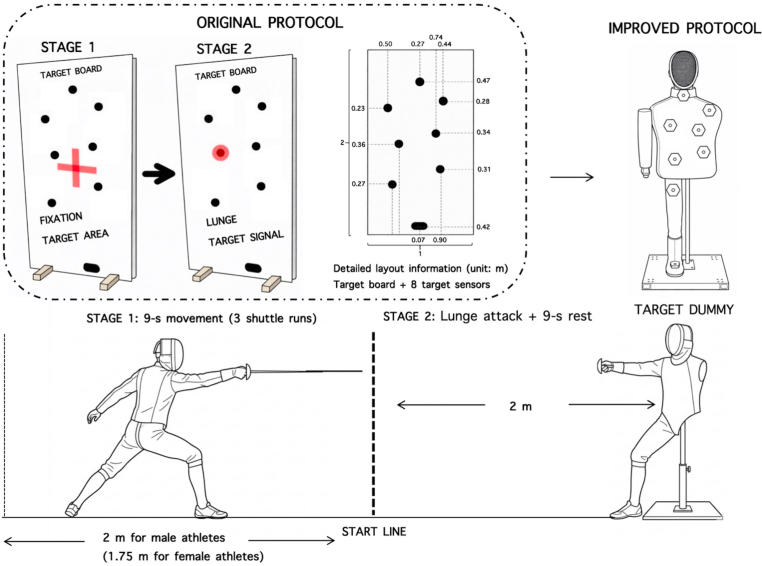
The illustration of the Epee fencing-specific test setting.

The study follows the test settings and instructions from Varesco et al. [4]. The FST consists of three shuttles (forward and backward) over 2m for men and 1.75m for women Epee fencers. This adjustment for the women's group has been deemed necessary based on the height difference. Shuttles must be completed within 9s. Participants will be taught to focus their attention on the eight flash-impact sensors (ReactionX Pro, China) attached to a fencer-shaped dummy (2 × 1m), as presented in Fig. 4. After the 9s shuttling, a green-colored sensor will be shown as the random lunge attack target. The target will undergo a randomization with replacement, that is, the same one could be presented multiple times. Once the target is presented, participants will be instructed to execute a lunge to deliver a valid hit on the middle of the sensor (i.e., a final attack to score) via the sword tip as quickly as possible. Furthermore, to better simulate the possible correction and reaction inhibition in real fencing, an incongruent cue will be presented in 10% of the trials [4]: a sensor will be flashed in red color for 300 ms before the real green target is shown. The incongruent cue will be presented close to the real target (i.e., within 40 cm) to mimic the real-world fast displacement in fencing. The trial will be separated by 9s for passive rest to simulate the temporal demands (i.e., work-to-rest ratio of 1:1) in Epee. Thus, each trial will last 18s. One FST will include 10 trials for 3min. The test outcomes will be the number of lunges hits on the real targets, hits on the wrong target, misses the targets, and the time from the moment each target is activated (i.e., lights up) to the moment it is hit or missed. Then, the average value of the lunge reaction time (LT) of the valid hits and the lunge attack accuracy (LAcc) will be calculated for analysis.

### 2.10 Data collection and management

The descriptive data, including participants' demographic information such as age, height, weight, and body mass index, self-reported injury history (i.e., injury site, time, stoppage period), current physical and mental health state, athletic level with training experience [25] (i.e., weekly training frequency, training years, highest competition level), coffee/smoking/sleep/diet behaviors (i.e., daily amount), educational status, academic courses and examination schedules in the upcoming study month, and currently registered competitions, will first be collected by an online questionnaire during familiarization.

All other quantitative outcome measures (i.e., subjective perceptions, performance variables, and HRmean) will be administered at two conditions, defined as EXP/CON_T0/1/2_. After data collection, participants' personal information will be completely deleted, except for the assigned exclusive identifier code (ID) to ensure pseudonymization. Data will be securely stored and confidentially managed according to the General Data Protection Regulation (GDPR) of 27 April 2016. The data collection and analysis will be monitored by the main investigator's three supervisors. The supervision group will make the final decision to terminate the study, if needed.

### 2.11 Statistical analyses

Data will be cleaned, analyzed, and visualized in MATLAB_R2023b software. The missing data will be reported and deleted by case. The data will be reported as mean and standard deviation. The significance level will be set at p < 0.05. Linear mixed-effect model (LMM) with fixed-effect factors for time, condition, and their interaction, and a random intercept for ID, will be applied to each performance variable (i.e., RT, RAcc, LT, LAcc) and subjective fatigue variable (i.e., MVAS, RPE). The Shapiro–Wilk test will be conducted to assess the normality of the residuals. Sphericity will be evaluated using Mauchly's test. When appropriate, Bonferroni post-hoc tests will be used. Other subjective perceptions (i.e., VAS-Re, VAS-Enj, NASA-TLX) and HRmean will be compared between conditions via paired sample t-tests. The normality of the difference scores will be verified using the Shapiro–Wilk test. If violated, the non-parametric Wilcoxon signed-rank test will be used instead. For significant effects in LMM, partial eta-squared will be calculated. For t-tests, Cohen's d will be computed. The classification criteria will follow Cohen's convention: for partial eta-squared, 0.01 = small, 0.06 = medium, and 0.14 = large effect; for d, 0.2 = small, 0.5 = medium, 0.8 = large effect.

## 3 Discussion

This study attempts to verify the previously documented advantages of VR technology[14,15] in a real-world competitive fencing context. The feasibility of VR will be explored to replace the human resources that a coach or sparring partner-led, competitive one-on-one combat requires during the pre-competition warm-up. We hypothesize that VR combat could serve as a warm-up for the subsequent fencing performance. Meanwhile, the brief VR combat is supposed to be flexible, self-organized, and entertaining to buffer fatigue perceptions [17].

This study design demonstrates strong novelty and practical relevance. First, the evidence supporting the redesign of the pre-competition warm-up is derived from empirical longitudinal monitoring data in official fencing competitions [8,9]. The VR application of the dual-task paradigm (combined physical and cognitive exercise) is promoted by positive findings from a 5-week brain endurance training intervention among fencers [18]. Furthermore, the structure, content, and timing of the warm-up session have been developed strictly based on empirical investigations in this fencing community. To the authors' knowledge, this is the first time VR will be integrated in a traditional fencing warm-up. By assessing subjective and objective indicators of mental fatigue and fencing-specific performance, this study will effectively bridge the gap between research and on-field fencing practice.

However, this study protocol is risked by several limitations that should be acknowledged. The acute effects of the intervention will not account for dose-related factors such as difficulty or duration of the VR utilization. Further research may be needed to optimize the dose and timing of the exergame. Moreover, the specialized warm-up will be evaluated using a skill test (i.e., FST) rather than in a real competition, which may limit the ecological validity of the findings [30]. Regardless, it provides a step towards a model for the assessment of performance. Despite these acknowledged constraints, the practice-oriented protocol will provide valuable insights into modulating pre-competition warm-up, managing mental fatigue, and enhancing fencing performance in a real-world context.

## Authorship contributions

Conceptualization: Chao Bian & Bart Roelands. Methodology: Chao Bian, Suzanna Russell, Špela Bogataj & Bart Roelands. Supervision: Suzanna Russell, Špela Bogataj & Bart Roelands. Writing - original draft: Chao Bian. Writing - review and editing: Chao Bian, Kevin De Pauw, Rafael Kons, Sidney Grosprêtre, Suzanna Russell, Špela Bogataj & Bart Roelands. All authors have approved the final version of this manuscript.

## Compliance with ethical standards

All authors declare that the study will be conducted in compliance with all ethical standards.

## Ethics approval

The study is approved by the ethics committee of Tsinghua University (THU-01-2026-0021).

## Data availability statement

No data will be available at this stage.

## Declaration of generative AI and AI-assisted technologies in the manuscript preparation process

No generative AI or AI-assisted technologies have been used in the manuscript preparation process.

## Funding

The study is funded by a tertiary research project at the University Medical Center Ljubljana (NO.20240208). Chao Bian is an awardee of the China Scholarship Council (NO.202208310018).

## Declaration of competing interest

All authors have no conflicts of interest relevant to this study.

## References

[1] Roi G.S., Bianchedi D. (2008). The science of fencing: implications for performance and injury prevention. Sports Med.

[2] Tarragó R., Bottoms L., Iglesias X. (2023). Temporal demands of elite fencing. PLoS One.

[3] Turner A.N., Kilduff L.P., Marshall G.J.G. (2017). Competition intensity and fatigue in elite fencing. J Strength Cond Res.

[4] Varesco G., Sarcher A., Doron J., Jubeau M. (2024). Striking a balance: exploring attention, attack accuracy and speed in fencing performance. Eur J Sport Sci.

[5] Gutiérrez-Davila M., Rojas F.J., Gutiérrez-Cruz C., Navarro E. (2019). Components of attack response inhibition in fencing: components of attack response inhibition in fencing. Eur J Sport Sci.

[6] Bian C., Russell S., De Pauw K., Habay J., Bogataj Š., Roelands B. (2025). Understanding of mental fatigue in elite fencing sports: perspectives from Chinese national level fencers. Front Psychol.

[7] Varesco G., Pageaux B., Cattagni T., Sarcher A., Martinent G., Doron J. (2023). Fatigue in elite fencing: effects of a simulated competition. Scand J Med Sci Sports.

[8] Bian C., Russell S., De Pauw K., Ampe T., Bogataj Š., Roelands B. (2025). Impacts of season phases and training variables on mental fatigue in real-world elite fencing. Int J Sports Physiol Perform.

[9] Bian C., Russell S., Kons R.L., Provyn S., Habay J., Bogataj Š. (2025). Evolution of mental fatigue with consecutive match progression in elite fencing. Int J Sports Physiol Perform.

[10] McGowan C.J., Pyne D.B., Thompson K.G., Rattray B. (2015). Warm-Up strategies for sport and exercise: mechanisms and applications. Sports Med.

[11] Holmberg P.M., Kelly V.G. (2025). Priming the conversation full circle: exploring mechanistic explanations for same-day performance effects following priming exercise stimuli. Int J Sports Physiol Perform.

[12] Díaz-García J., Rubio-Morales A., Manzano-Rodríguez D., García-Calvo T., Ring C. (2025). Cognitive priming during Warmup enhances sport and exercise performance: a goldilocks effect. Brain Sci.

[13] Günar B.B., Bavlı Ö. (2025). Effectiveness of virtual reality games on the specific sport skills of adolescents. BMC Sports Sci Med Rehabil.

[14] Kittel A., Lindsay R., Le Noury P., Wilkins L. (2024). The use of extended reality technologies in sport perceptual-cognitive skill research: a systematic scoping review. Sports Med Open.

[15] Connolly J., Alder D., Frame M., Wilson A.D. (2025). Training decision making in sports using virtual reality: a scoping review. Int Rev Sport Exerc Psychol.

[16] Howard M.C., Gutworth M.B., Jacobs R.R. (2021). A meta-analysis of virtual reality training programs. Comput Hum Behav.

[17] Grosprêtre S., Marcel-Millet P., Eon P., Wollesen B. (2023). How exergaming with virtual reality enhances specific cognitive and visuo-motor abilities: an explorative Study. Cogn Sci.

[18] Varesco G., Staiano W., Bracco M., Pageaux B., Soulas L., Goisbault M. (2025). Effects of 5-Week brain endurance training on fatigue and performance in elite youth epée fencers. Int J Sports Physiol Perform.

[19] Fadeev K.A., Smirnov A.S., Zhigalova O.P., Bazhina P.S., Tumialis A.V., Golokhvast K.S. (2020). Too Real to be virtual: autonomic and EEG responses to extreme stress scenarios in virtual reality. Behav Neurol.

[20] Grosprêtre S., Mathiot J., Eon P., Ruffino C. (2025). Covariation of corticospinal excitability and the autonomous nervous system by virtual reality: the roller coaster effect. Exp Brain Res.

[21] Csathó Á., Van der Linden D., Matuz A. (2024). Change in heart rate variability with increasing time-on-task as a marker for mental fatigue: a systematic review. Biol Psychol.

[22] Ochi G., Kuwamizu R., Fujimoto T., Ikarashi K., Yamashiro K., Sato D. (2022). The effects of acute virtual reality exergaming on mood and executive function: exploratory crossover trial. JMIR Serious Games.

[23] Schampheleer E., Habay J., Proost M., Arauz Y.L.A., Russell S., Roose M. (2025). Current practices for mental fatigue quantification and induction in movement Science: introducing the SPeCIFY Guidelines. Sports Med.

[24] Harris D.J., Buckingham G., Wilson M.R., Brookes J., Mushtaq F., Mon-Williams M. (2020). The effect of a virtual reality environment on gaze behaviour and motor skill learning. Psychol Sport Exerc.

[25] McKay A., Stellingwerff T., Smith E., Martin D., Mujika I., Goosey-Tolfrey V. (2021). Defining training and performance caliber: a participant classification framework. Int J Sports Physiol Perform.

[26] Russell S., Jenkins D., Rynne S., Halson S., Kelly V. (2019). What is mental fatigue in elite sport? Perceptions from athletes and staff. Eur J Sport Sci.

[27] Turner A.N., Buttigieg C., Marshall G., Noto A., Phillips J., Kilduff L. (2017). Ecological validity of the session rating of perceived exertion for quantifying internal training load in fencing. Int J Sports Physiol Perform.

[28] Doron J., Lienhart N., Martinent G., Goisbault M. (2024). Coping with intensive training demands: a longitudinal investigation of the relationships between appraisal, emotion, coping effectiveness and engagement among elite fencers. Int J Sport Exerc Psychol.

[29] Basner M., Mollicone D., Dinges D.F. (2011). Validity and sensitivity of a Brief Psychomotor Vigilance Test (PVT-B) to total and partial sleep deprivation. Acta Astronaut.

[30] Bian C., Russell S., Mali A., Lathouwers E., De Pauw K., Habay J. (2025). Methodological considerations and effectiveness for ecologically valid mental fatigue inducement in sports: a systematic review. Sports Med Open.

